# Application of intraarterial superselective indocyanine green angiography in bypass surgery for adult moyamoya disease

**DOI:** 10.3389/fneur.2023.1241760

**Published:** 2023-10-16

**Authors:** Haojin Ni, Yiwen Wu, Chenhui Zhou, Xianru Li, Shengjun Zhou, Wenting Lan, Zhimeng Zhang, Yi Huang, Haifeng Wang, Jinghui Lin

**Affiliations:** ^1^Department of Neurosurgery, The First Affiliated Hospital of Ningbo University, Ningbo, China; ^2^Department of Radiology, The First Affiliated Hospital of Ningbo University, Ningbo, China

**Keywords:** moyamoya disease, cerebral revascularization, intravenous ICG video-angiography, intra-arterial ICG video-angiography, blood flow direction, cortical perfusion range

## Abstract

**Background:**

Extracranial-intracranial (EC-IC) bypass surgery is the main treatment approach to moyamoya disease, and an accurate assessment of the patency of anastomosis is critical for successful surgery. So far, the most common way to do this is the intraoperative intravenous indocyanine green (ICG) video-angiography. Intra-arterial ICG-VA has been applied to treat peripheral cerebral aneurysms, spinal arteriovenous fistulas, and dural arteriovenous fistulas, but few reports have concerned the use of arterial injection of ICG to evaluate anastomotic patency. This research aims to explore the feasibility and effects of catheter-guided superficial temporal artery injection of ICG in the evaluation of anastomotic patency after bypass surgery.

**Methods:**

In this study, 20 patients with moyamoya disease or syndrome who underwent bypass surgery were divided into two groups, one who received intravenous ICG angiography and the other who received intra-arterial ICG angiography, to compare the two injection methods for vascular anastomosis patency. We conducted conventional intraoperative digital subtraction angiography (DSA) in a hybrid operating room during extracranial-intracranial (EC-IC) bypass surgery, including the additional step of injecting ICG into the main trunk of the superficial temporal artery (STA) through a catheter.

**Results:**

Intra-arterial injection of indocyanine green video-angiography (ICG-VA) indicated good patency of the vascular anastomosis when compared with conventional digital subtraction angiography (DSA) and intravenous ICG-VA, confirming the feasibility of using the arterial injection of ICG for assessing anastomotic patency. And intra-arterial ICG-VA results in faster visualization than intravenous ICG-VA (*p* < 0.05). Besides, ICG-VA through arterial injection provided valuable information on the vascular blood flow direction after the bypass surgery, and allowed for visual inspection of the range of cortical brain supply from the superficial temporal artery and venous return from the cortex. Moreover, arterial injection of ICG offered a rapid dye washout effect, reducing the repeat imaging time.

**Conclusion:**

This study indicates that intra-arterial ICG-VA has good effects in observing the direction of blood flow in blood vessels and the range of cortical brain supply from the STA, which reflects blood flow near the anastomosis and provides additional information that may allow the postoperative prediction of cerebral hyperperfusion syndrome. However, the procedure of intra-arterial ICG-VA is relatively complicated compared to intravenous ICG-VA.

## 1. Introduction

Moyamoya disease is a cerebrovascular disease that mainly manifests in the distal internal carotid artery, as well as the proximal anterior and middle cerebral arteries which are progressively stenosed or occluded, leading to the formation of an abnormal vascular network at the base of the brain (1). It primarily occurs in East Asian countries like Japan, China, etc. The major complications of moyamoya disease include transient ischemic attack (TIA), ischemic and hemorrhagic strokes, cognitive impairment, headaches, and seizures, which can lead to disability or even death (2). So far, there are no definitely effective drugs for the treatment of moyamoya disease, and further investigations are needed (3). Surgical revascularization surgery includes direct, indirect, and combined bypass surgeries. Meta-analysis suggests that the first and the third have significant advantages in treating late-stage stroke and bleeding patients (4). As direct and combined bypass surgeries are the main treatments of moyamoya disease, the assessment of the patency of the anastomosed vessels during surgical bypass of moyamoya disease is therefore critical to ensuring the surgical success.

In 2003, Raabe et al. (5) introduced indocyanine green video-angiography (ICG-VA), which has since then been extensively used for the evaluation of cerebral blood flow intraoperatively in procedures such as aneurysm clipping, bypass surgery, arteriovenous malformation (AVM) and arteriovenous fistula (AVF) (6). In bypass surgery, the evaluation of patency at the anastomotic site is mainly achieved by intravenous injection of ICG (7). However, intravenous injection of ICG has several drawbacks. For instance, due to dilution by the circulation, the intracranial arteries need a large amount of ICG after peripheral venous injection of indocyanine green, and the metabolic process in cerebral vessels after imaging has to last 15 min (8) and is even unrepeatable within a short time. Since synchronization easily occurs at the anastomotic vessels when the indocyanine green flows to the cortical vessels through the carotid artery, the contrast in the imaging area is not abundantly clear, which affects the afterward assessment of results. Currently, although arterial injection of ICG has been applied to treat peripheral cerebral aneurysms, spinal arteriovenous fistulas, and dural arteriovenous fistulas (9–11), there have been few reports about the use of superselective arterial injection of ICG to estimate the patency of the anastomotic site during bypass surgery (12). Thus, this study attempts to estimate the patency of the anastomotic site after cerebral blood flow reconstruction by superselective injection of ICG into the superficial temporal artery through a catheter (select right femoral artery and apply a 5F curved catheter), and investigate the feasibility and effects of this method.

## 2. Methods

### 2.1. Patients and acquisition of data

This study involved 20 patients diagnosed with moyamoya disease or syndrome and with indications for surgery, all of whom underwent preoperative digital subtraction angiography (DSA) imaging evaluation based on the diagnostic criteria for moyamoya diseases in the 2021 Japanese moyamoya disease management guidelines (13). The inclusion criteria for patients are as follows:

- Male or female subjects, age ranging between 18 and 70 years.
- Digital subtraction angiography (DSA) imaging demonstrating stenosis or occlusion in the arteries centered on the terminal portion of unilateral or bilateral ICA and abnormal vascular networks in the vicinity of the stenotic or occlusive lesions in the arterial phase.
- A qualifying transient ischemic attack (TIA) or ischemic stroke or hemorrhagic stroke in the stenotic or occlusive territory must have occurred within the past 12 months.
- No previous history of EC-IC bypass surgery.
- Must be competent to give informed consent.

These patients were admitted to The First Affiliated Hospital of Ningbo University between December 2022 and April 2023, and underwent STA-MCA bypass surgery combined with intraoperative cerebral angiography. The research protocol has been ratified by the Medical Ethics Committee of the First Affiliated Hospital of Ningbo University (2022-085A), conducted according to relevant organizational guidelines, and complied with the Helsinki Declaration (revised in 1983). We obtained written informed consent from all the patients. Preoperative and postoperative radiographic data were obtained from the department’s digital management software, and videos recorded with a KINEVO 900 microscope (Carl Zeiss) were analyzed and evaluated. Numerical data were compared within the same group using the paired *t*-test. All analyses were performed with IBM SPSS Statistics 25.0. Results with *p* < 0.05 were considered significant.

### 2.2. Surgical procedure

All STA-MCA bypass surgeries and intraoperative cerebral angiography were executed, respectively, by the same experienced neurosurgeon in the hybrid operating room. Following general anesthesia, the patient was in a supine position with the head tilted 60° to the opposite side. The perineum was disinfected and draped, the Seldinger technique was used to pierce the right femoral artery, a 5F sheath was inserted, and a 5F curved catheter was selected and advanced to the beginning of a parietal branch of the superficial temporal artery on the affected side. The catheter position was maintained, and heparinized saline was continuously infused through the catheter and manually flushed every 20 min to prevent thromboembolism. Next, the head was disinfected and draped, a curved incision of approximately 15 cm was made on the ipsilateral frontotemporal area, and the scalp was incised to create a flap. The temporal muscle was partially incised to create a muscle flap, and the muscle was dissected and flipped forward with adequate intraoperative protection of the superficial temporal artery. Based on the preoperative digital subtraction angiography, the bone flap of approximately 6 cm × 8 cm was removed to avoid the middle meningeal artery. Then, the dura mater was suspended, and the parietal branch of the superficial temporal artery was separated and the dura mater was radially incised. The brain surface was examined, and a suitable receptor vessel was selected for the STA-MCA anastomosis. After the anastomosis, the fluorescence microscope was turned on, and 25 mg indocyanine green (25 mg in 5 cc) was intravenously injected peripherally to determine the patency of the anastomosis. In the ICG video-angiography, the imaging is recorded under a microscope while ICG is injected. Subsequently, digital subtraction angiography (DSA) was conducted to evaluate the blood flow patency of the anastomotic site. Afterward, indocyanine green was intra-arterially injected through the catheter, and the anastomotic site was evaluated using an integrated ICG-VA microscope. The catheter sheath was removed, the vascular closure device was used to close the vessel, and local hemostatic dressings were applied. The temporal muscle was repaired, and the dural defect was closed in layers. Finally, conventional postoperative computed tomography (CT) scans were performed 2 hours after surgery to detect any secondary cerebral hemorrhage. In addition, it is necessary to monitor the patient’s systemic symptoms and to check the magnetic resonance imaging (MRI) immediately if there are signs of cerebral infarction.

### 2.3. Intraoperative indocyanine green videoangiography

A microscope (KINEVO 900, Carl Zeiss), outfitted with a fluorescence illuminant (wavelength 700–850 nm) and an infrared camera for imaging, was used to integrate near-infrared indocyanine green fluorescence imaging. It provides an automatic zoom function and adjusts the camera gain automatically during ICG-VA to the near-infrared signal strength within the camera’s dynamic range, achieving optimal visualization of the fluorescent area. The microscope was placed vertically about 300 mm away from the study area. During ICG-VA, the surgical area light was turned off, and 25 mg of indocyanine green was dissolved in 5 mL of saline and administered as a single intravenous injection through a peripheral venous catheter. When reaching the corresponding area, ICG emits fluorescence after being excited by near-infrared light, then converted to a black-white image which is displayed on the microscope monitor. The operator evaluated the patency of the anastomosis site by looking at the ICG videoangiography recording on the microscope monitor. Similarly, the graft patency of the anastomosis site was evaluated by observing the fluorescence image through the microscope after ICG was injected into the superficial temporal artery through a catheter. 25 mg of indocyanine green in 5 mL of saline was dissolved and a smaller dose (0.5 mL) was used to compare the results after peripheral venous injection. Flow control at 2 mL/min is required by micropump injection.

## 3. Results

### 3.1. Demographics

A total of 20 patients with MMD who underwent STA-MCA anastomosis surgery were included in this study, including 12 males and 8 females. The patients’ ages ranged from 23 to 70 years (mean age 51 years). The patency of the anastomosis was evaluated using both intravenous and arterial injection of ICG-VA in all the patients and verified by intraoperative DSA. Among them, 19 were detected with symptoms of transient ischemic attacks, ischemic strokes, or hemorrhagic strokes, except one being asymptomatic, as shown in Table 1.

**Table 1:** Study participants clinical characteristics.

Case No.	Sex	Age	Symptoms	Operated side
1	Female	53	Ischemia	Left
2	Male	43	TIA	Right
3	Female	23	Hemorrhage	Right
4	Male	42	Headache	Left
5	Male	40	Hemorrhage	Left
6	Female	37	Hemorrhage	Right
7	Female	58	Ischemia	Left
8	Male	53	Headache	Left
9	Female	57	Ischemia	Right
10	Male	56	Ischemia	Right
11	Male	55	Ischemia	Right
12	Male	59	TIA	Left
13	Male	70	TIA	Left
14	Male	47	Hemorrhage	Right
15	Female	66	Ischemia	Left
16	Male	64	TIA	Left
17	Male	31	Hemorrhage	Right
18	Male	63	Ischemia	Left
19	Female	50	TIA	Left
20	Female	47	None	Right

### 3.2. Assessment of anastomotic patency

**Figure 1:**
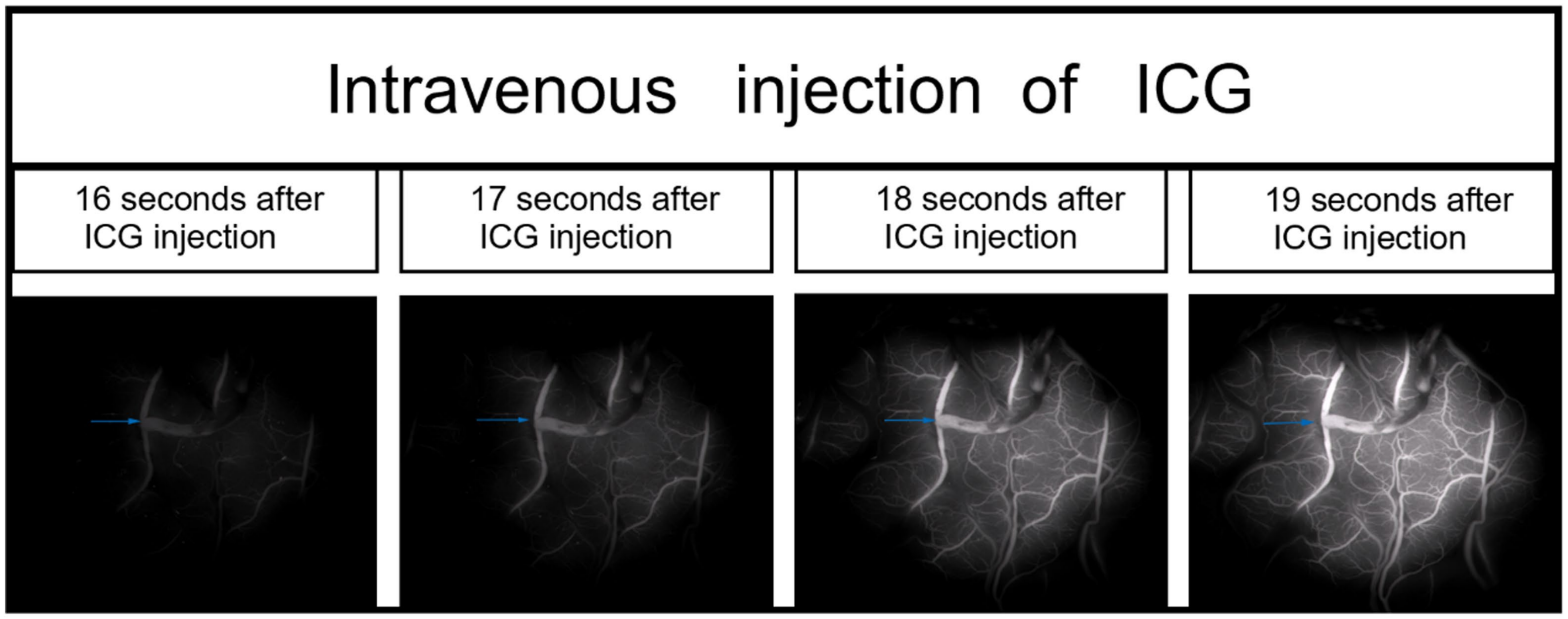
During the right middle cerebral artery (M4) bypass surgery, intravenous indocyanine green (ICG) video-angiography was performed to visualize blood vessels in one case (the anastomotic site is indicated by the blue arrow).

**Figure 2:**
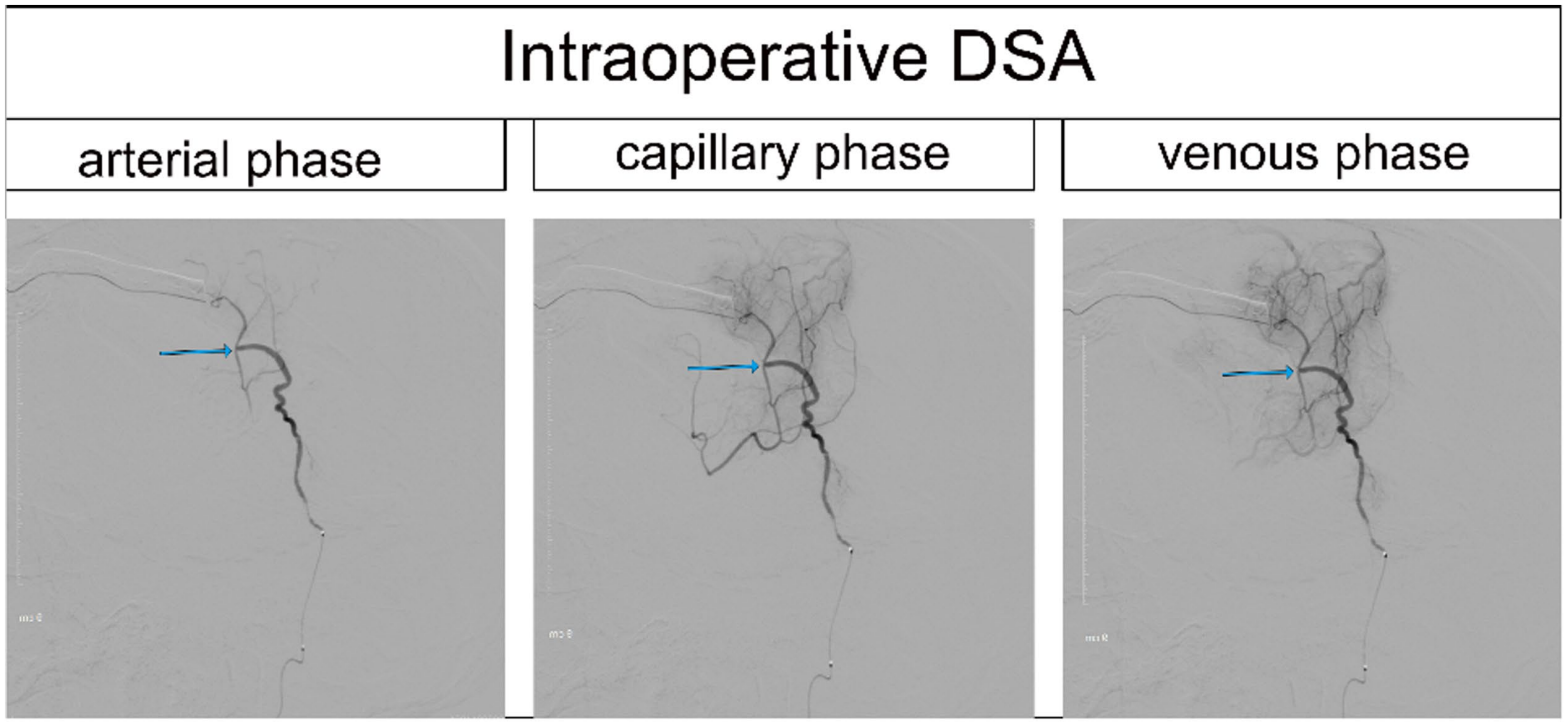
Intraoperative DSA of the same patient (the anastomotic site is indicated by the blue arrow).

**Figure 3:**
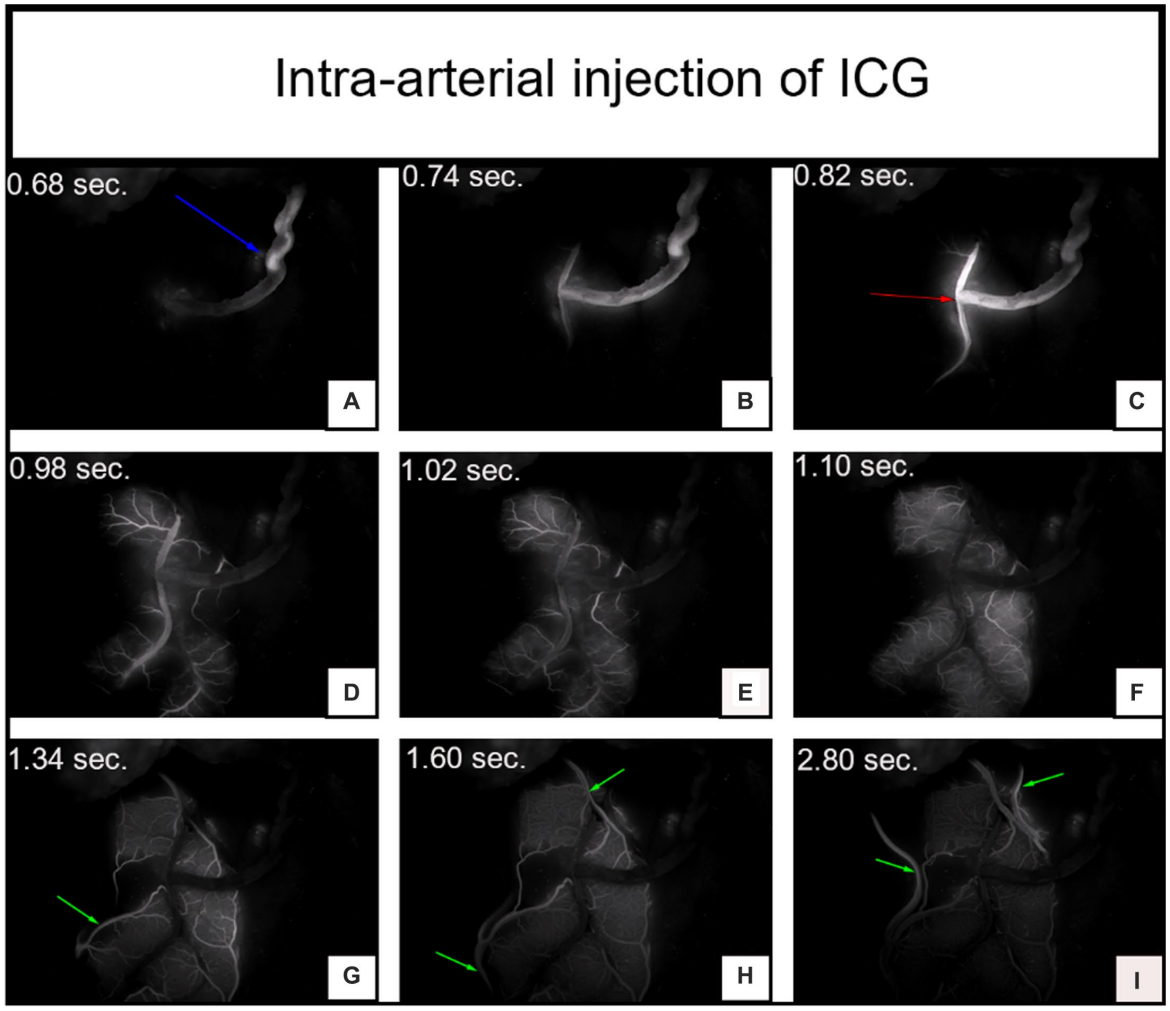
The video angiography results of the same patient after arterial injection of indocyanine green (ICG). Panels **(A–C)** depict the arterial phase of ICG injection, where the blue arrow indicates the temporal superficial artery and the red arrow indicates the anastomosis site. Panels **(D–F)** represent the capillary phase of ICG injection. Panels **(G-I)** display the venous phase, where the green arrow indicates the refluxing vein. The time of ICG signal after injection is noted in the upper left corner.

Each patient received intravenous injection of ICG-VA, followed by intraoperative DSA and intra-arterial injection of ICG-VA. Based on the visualized demonstration of ICG after intravenous injection in Figure 1 and the evaluation results of intraoperative DSA for the same patient in Figure 2, the patency of the anastomotic vessel is clear, which is also verified by the evaluation results during intra-arterial injection of ICG-VA in the arterial phase of the same patient shown in Figures 3A–C. It should be noted that there were no adverse reactions to indocyanine green during the operation. Thus, the comparison of the results between intra-arterial injection and the intravenous injection and their intraoperative DSA evaluation all confirmed the effectiveness of intra-arterial injection of ICG-VA in evaluating anastomosis patency.

### 3.3. Timing of ICG signal after injection

In our current study, we recorded the timing of ICG signals after injection by both methods. As shown in Table 2, we counted the time required for two methods of ICG video-angiography to begin imaging. The average time required for intravenous injection of ICG video-angiography was 27.3 s, while the average time required for intra-arterial injection of ICG video-angiography was only 1.08 s, there is a significant difference between the two data, it can be concluded that intra-arterial ICG-VA results in faster visualization than intravenous ICG-VA(*p* < 0.05).

**Table 2:** The time from ICG injection to ICG signal appearance (seconds).

Case No.	Intravenous ICG-VA	Intra-arterial ICG-VA	*p*-value
1	19	0.1	
2	22.7	0.8	
3	23.1	2.2	
4	25.4	0.5	
5	26.2	0.2	
6	16	0.6	
7	50	3.2	
8	21.8	1.2	
9	34.4	1.2	
10	37.6	1.6	
11	20.5	1.2	
12	22.2	0.3	
13	29.2	1.2	
14	22	0.2	
15	37.2	2.2	
16	25.2	0.5	
17	26.7	1.2	
18	29.8	0.8	
19	33.6	2	
20	23.4	0.4	
Mean	27.3	1.08	2 × 10^−12^

### 3.4. Blood flow observations with intravenous and intra-arterial ICGA

**Figure 4:**
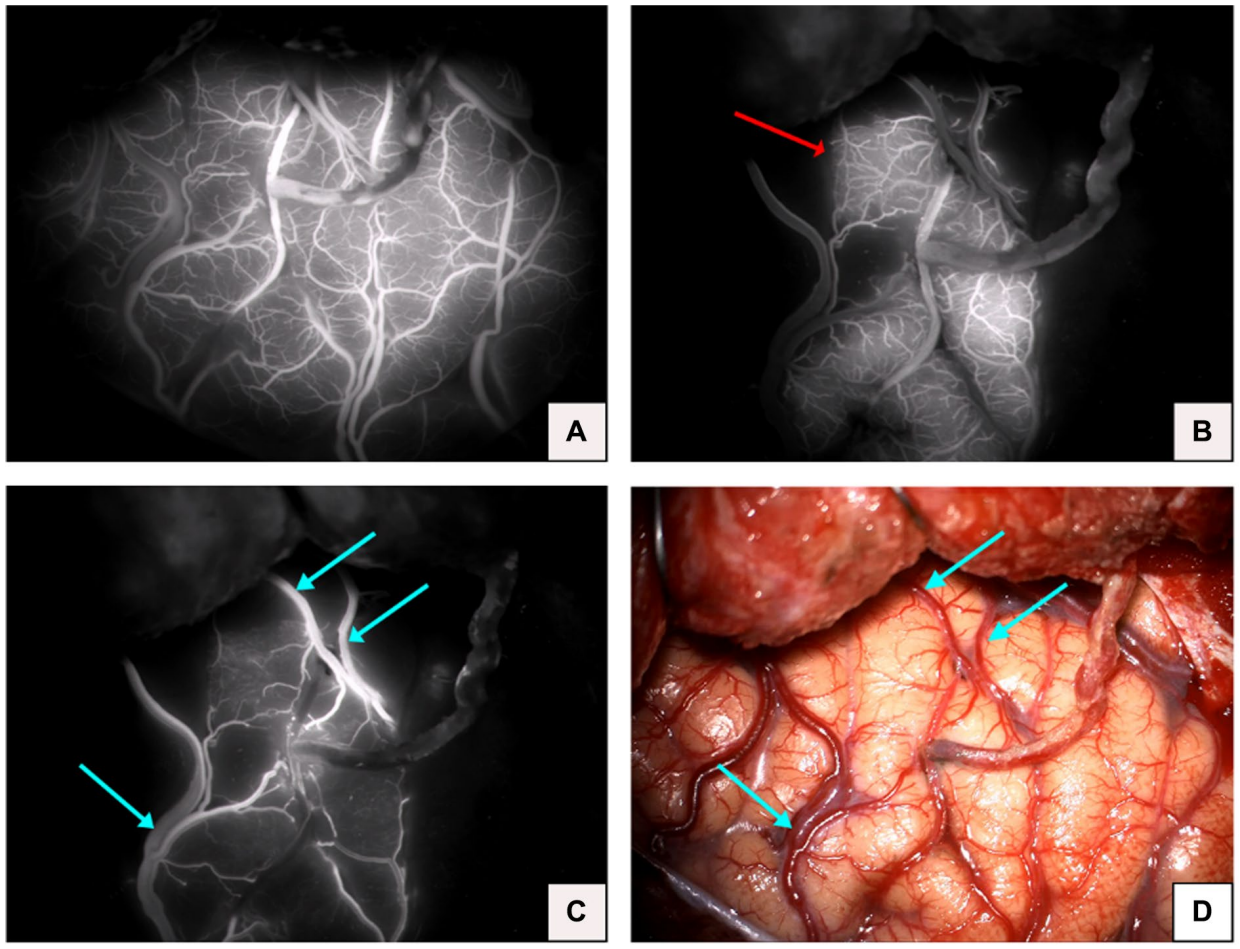
**(A)** Image of complete visualization after intravenous injection of ICG. **(B)** Microvascular phase after arterial injection of ICG in the same patient (red arrow indicates the cortical area of the brain supplied by the superficial temporal artery). **(C)** Venous phase after arterial injection of ICG in the same patient (blue arrow indicates the venous return). **(D)** Microscopic view of the operative field after completion of the bypass in the same patient (blue arrow indicates venous return).

Although fluorescence is emitted near the anastomosis site, as shown in the imaging after intravenous injection of ICG in Figure 1, which makes it difficult to evaluate the blood flow direction after bypass grafting, the superficial temporal artery is immediately visible and subsequently flows into the recipient vessel, cerebral capillaries and refluxing vein after intra-arterial injection of ICG, as shown in Figure 3, which clearly illustrates the arterial, capillary, and venous phases of ICG entering the vessels. Furthermore, the approximate range of the cerebral cortex is supplied by the superficial temporal artery, as shown in Figure 4B, and there exists the venous reflux of the cortical area with the corresponding vein being identifiable, as shown in Figures 4C,D. However, due to the low contrast, ICG-VA intravenously injected fails to demonstrate the above positive effects, according to Figure 4A. Comparing Figure 4B and Figure 4C, we found that despite the difference of only 5 s between the two images, most of the ICG in the capillary phase was not visible after entering the venous phase, indicating that the dye washout effect of arterial injection of ICG is stronger.

## 4. Discussion

Direct and combined bypass are currently the main methods for treating adult moyamoya disease (4), and vascular anastomosis is the key to surgery. Due to the slender nature of the recipient and donor vessels, vascular anastomosis is extremely difficult, and intraoperative excessive damage to the intima is more likely to cause acute thrombosis (14) and occlusion of the anastomosis. Failing a timely warning and possible early detection can produce iatrogenic cerebral infarction and new-onset neurological dysfunction (7). Rapid and accurate evaluation of the patency of the anastomotic site during the bypass surgery thus becomes critical for the success of the surgery.

So far, there are various methods for intraoperative evaluation of vascular anastomosis patency, and among them, ICG video-angiography is the most common and standard technique for assessing anastomosis patency during bypass surgery, owing to its good temporal and spatial resolution and ease of use. Previously, peripheral venous injection of ICG was usually used to evaluate vascular anastomosis, while for our treatment, the images are instantly generated and visible on the screen during intra-arterial injection of ICG, while for the conventional peripheral intravenous injection, the process of imaging needs 10–30 s (15), in our study, the average time required for intravenous injection of ICG video-angiography was 27.3 s, which is consistent with the above. After imaging, our method of the intra-arterial injection allows the direct observation of blood flow direction, which makes the result more objective, reflecting the patency of the anastomotic site and visually demonstrating that the superficial temporal artery is supplying blood to the recipient vessel, representing the success of this bypass surgery. But for the conventional injection of ICG angiography, there is an uncertainty about the direction of cerebral blood flow after grafting and difficulty in detecting the blood flow direction when the cerebral blood flow velocity is high, since it requires an in-depth analysis and detection under the FLOW 800 system (16) with the ROI in a better position as setting, ultimately causing a delay and some deviation. Furthermore, in our study, it becomes easily identifiable of the cortical area supplied by the superficial temporal artery and the venous reflux of the cortical area. Currently, cerebral hyperperfusion syndrome (CHS) is a not rare and thorny complication after bypass surgery, mainly manifested as epilepsy, headache, and even cerebral hemorrhage (17). A study indicates that the larger the direct perfusion range of the superficial temporal artery, the lesser the possibility of CHS occurring (18), and a case report prompts that cortical venous reddening near the anastomotic site after bypass surgery may be a sign of hyperperfusion (19). Therefore, although there are many factors that affect the occurrence of CHS, the observation of the supply area of the superficial temporal artery and cortical venous reflux can serve as additional information that may allow the prediction of cerebral hyperperfusion syndrome for subsequent prevention and treatment. Additionally, for the conventional method, the flowing of intravenous ICG into the cortical vessels through the internal carotid artery probably causes almost simultaneous imaging of the vessels in the field of surgery, which results in poorer image contrast.

The use of a relatively small dose of ICG during this arterial injection process also obtains a favorable imaging result. Although very few reports of adverse reactions to ICG but some cases exposing severe allergic reactions and cardiac arrest (20), therefore a smaller amount of ICG is conducive to the reduction of the incidence of adverse reactions (21). In addition, intra-arterial injection has a better dye washing effect than intravenous injection (22). Moreover, the reduced dosage of ICG results in less residual time in cerebral blood vessels, and in case of poor imaging effect during the operation, the next imaging can be performed more quickly, shortening the surgery time (according to our experience, usually about 1 min of the interval between two vascular imaging).

DSA is considered the gold standard for diagnosing moyamoya disease and assessing the patency of bypass surgery (23). In our study, apart from conventional cerebral angiography, catheter superselection, and indocyanine green angiography into the superficial temporal artery were also employed to obtain images so that these images were congruent with those observed under the surgical microscope, allowing for a better understanding of the anatomical structures with higher spatial resolution. Moreover, satisfactory imaging results were also achieved when injection of ICG into the branch vessel of STA using a syringe after STA-MCA anastomosis, which was specially designed for some patients who refused DSA (24).

Intraoperative microvascular Doppler ultrasound is proposed to be performed directly on the bypass vessel to assess patency using quantitative vascular flow parameters (15). A retrospective study of 51 patients with obstructive cerebrovascular disease who underwent bypass surgery suggested that a cutoff flow index (CFI) of 0.5 can be used as a threshold for evaluating graft patency, with a graft patency rate of 92% for cases with CFI >0.5 and 50% for cases with CFI <0.5 (25). However it should be noted that in view of a high requirement of Doppler technique for the angle between the probe and the vessel, measurements at different angles will yield significantly different results. Besides, it is not as perfect as needed in the spatial resolution and image quality, posing an insuperable obstacle to the evaluation of blood flow in tiny vessels (26). By contrast, intracarotid injection of indocyanine green through a catheter enables more direct observation of the patency of the anastomosis and the filling status of the cortical small vessels.

This study has some limitations. First, intra-arterial injection of ICG is not covered by the FDA permission. However, injection of a lower dose of ICG should be acceptable, compared with intravenous injection. Second, the sample capacity of this study is comparatively small. More data and cases will be better for comparative research to thoroughly evaluate the security and availability of this technology.

## 5. Conclusion

In vascular bypass anastomosis for moyamoya disease, intra-arterial ICG video-angiography contributes to achieving the same evaluation effect as intravenous ICG video-angiography for evaluating the patency of the anastomotic site and giving a visual display of the blood flow direction of the bypass vessel. In addition, during the imaging process, the cortical area approximately supplied by the donor vessels is observable, providing additional information that may allow the prediction of postoperative cerebral hyperperfusion syndrome. However, the procedure of intra-arterial ICG-VA is relatively complicated compared to intravenous ICG-VA.

## Data availability statement

The raw data supporting the conclusions of this article will be made available by the authors, without undue reservation.

## Ethics statement

The studies involving humans were approved by Medical Ethics Committee of the First Affiliated Hospital of Ningbo University (2022-085A). The studies were conducted in accordance with the local legislation and institutional requirements. The participants provided their written informed consent to participate in this study. Written informed consent was obtained from the individual(s) for the publication of any potentially identifiable images or data included in this article.

## Author contributions

JL: conceptualization, project administration, writing—review and editing, funding acquisition, and supervision. XL and SZ: methodology. HN and CZ: software and investigation. WL and ZZ: validation. YH and HW: formal analysis. HN and YW: writing—original draft preparation. All authors contributed to the article and approved the submitted version.

## References

[1] Scott R Smith E. Moyamoya disease and moyamoya syndrome. N Engl J Med. (2009) 360:1226–37. doi: 10.1056/NEJMra0804622 19297575

[2] Shang S Zhou D Ya J Li S Yang Q Ding Y. Progress in moyamoya disease. Neurosurg Rev. (2020) 43:371–82. doi: 10.1007/s10143-018-0994-5 29911252

[3] Zhang X Xiao W Zhang Q Xia D Gao P Su J. Progression in moyamoya disease: clinical features, neuroimaging evaluation, and treatment. Curr Neuropharmacol. (2022) 20:292–308. doi: 10.2174/1570159X19666210716114016, PMID: 34279201 PMC9413783

[4] Nguyen VN Motiwala M Elarjani T Moore KA Miller LE Barats M. Direct, indirect, and combined extracranial-to-intracranial bypass for adult moyamoya disease: an updated systematic review and meta-analysis. Stroke. (2022) 53:3572–82. doi: 10.1161/STROKEAHA.122.039584, PMID: 36134563

[5] Raabe A Beck J Gerlach R Zimmermann M Seifert V. Near-infrared indocyanine green video angiography: a new method for intraoperative assessment of vascular flow. Neurosurgery. (2003) 52:132–9. doi: 10.1227/00006123-200301000-00017, PMID: 12493110

[6] Marchese E Della Pepa GM La Rocca G Albanese A Ius T Simboli GA. Application of indocyanine green video angiography in vascular neurosurgery. J Neurosurg Sci. (2019) 63:656–60. doi: 10.23736/S0390-5616.19.04753-2, PMID: 31339116

[7] Ambekar S Babu A Pandey P Devi IB. Intraoperative assessment of STA-MCA bypass patency using near-infrared indocyanine green video-angiography: a preliminary study. Neurol India. (2012) 60:604–7. doi: 10.4103/0028-3886.105194 23287322

[8] Shimada K Yamaguchi T Miyamoto T Sogabe S Korai M Okazaki T. Efficacy of intraarterial superselective indocyanine green videoangiography in cerebral arteriovenous malformation surgery in a hybrid operating room. J Neurosurg. (2020) 134:1544–52. doi: 10.3171/2020.3.JNS20319, PMID: 32442970

[9] Gruber A Dorfer C Bavinzski G Standhardt H Ferraz-Leite H Knosp E. Superselective indocyanine green angiography for selective revascularization in the management of peripheral cerebral aneurysms. AJNR Am J Neuroradiol. (2012) 33:E36–7. doi: 10.3174/ajnr.A2424, PMID: 21415146 PMC7966454

[10] Horie N So G Debata A Hayashi K Morikawa M Suyama K. Intra-arterial indocyanine green angiography in the management of spinal arteriovenous fistulae: technical case reports. Spine. (2012) 37:E264–7. doi: 10.1097/BRS.0b013e31822ba834 21738090

[11] Sasaki K Endo H Niizuma K Nishijima Y Osawa S Fujimura M. Efficacy of intra-arterial indocyanine green angiography for the microsurgical treatment of dural arteriovenous fistula: a case report. Surg Neurol Int. (2020) 11:46. doi: 10.25259/SNI_588_2019, PMID: 32257572 PMC7110105

[12] Simal-Julian JA Miranda-Lloret P Evangelista-Zamora R Sanroman-Alvarez P Perez De San Roman L Perez-Borreda P. Indocyanine green videoangiography methodological variations: review. Neurosurg Rev. (2015) 38:49–57. doi: 10.1007/s10143-014-0570-6 25171963

[13] Kuroda S Fujimura M Takahashi J Kataoka H Ogasawara K Iwama T. Diagnostic criteria for moyamoya disease—2021 revised version. Neurol Med Chir. (2022) 62:307–12. doi: 10.2176/jns-nmc.2022-0072, PMID: 35613882 PMC9357455

[14] Mikami T Suzuki H Ukai R Komatsu K Akiyama Y Wanibuchi M. Predictive factors for acute thrombogenesis occurring immediately after bypass procedure for moyamoya disease. Neurosurg Rev. (2020) 43:609–17. doi: 10.1007/s10143-019-01086-4, PMID: 30767097

[15] Cavallo C Gandhi S Zhao X Belykh E Valli D Nakaji P. Applications of microscope-integrated Indocyanine green videoangiography in cerebral revascularization procedures. Front Surg. (2019) 6:59. doi: 10.3389/fsurg.2019.00059, PMID: 31850362 PMC6902023

[16] Murai Y Nakagawa S Matano F Shirokane K Teramoto A Morita A. The feasibility of detecting cerebral blood flow direction using the indocyanine green video angiography. Neurosurg Rev. (2016) 39:685–90. doi: 10.1007/s10143-016-0726-7, PMID: 27136915

[17] Zhao WG Luo Q Jia JB Yu JL. Cerebral hyperperfusion syndrome after revascularization surgery in patients with moyamoya disease. Br J Neurosurg. (2013) 27:321–5. doi: 10.3109/02688697.2012.757294 23461748

[18] Yang D Zhang X Tan C Han Z Su Y Duan R. Intraoperative transit-time ultrasonography combined with FLOW800 predicts the occurrence of cerebral hyperperfusion syndrome after direct revascularization of moyamoya disease: a preliminary study. Acta Neurochir. (2021) 163:563–71. doi: 10.1007/s00701-020-04599-w, PMID: 33006072

[19] Machida T Ono J Nomura R Fujikawa A Nagano O Higuchi Y. Venous reddening as a possible sign of hyperperfusion after superficial temporal artery-middle cerebral artery anastomosis for moyamoya disease: case report. Neurol Med Chir. (2014) 54:827–31. doi: 10.2176/nmc.cr.2013-0261 PMC4533381 24670309

[20] Hope-Ross M Yannuzzi LA Gragoudas ES Guyer DR Slakter JS Sorenson JA. Adverse reactions due to indocyanine green. Ophthalmology. (1994) 101:529–33. doi: 10.1016/S0161-6420(94)31303-0 8127574

[21] Speich R Saesseli B Hoffmann U Neftel K Reichen J. Anaphylactoid reactions after indocyanine-green administration. Ann Intern Med. (1988) 109:345–6. doi: 10.7326/0003-4819-109-4-345_2 3395048

[22] Yoshioka H Kinouchi H Nishiyama Y Kanemaru K Yagi T Hanihara M. Advantage of microscope integrated for both indocyanine green and fluorescein videoangiography on aneurysmal surgery: case report. Neurol Med Chir. (2014) 54:192–5. doi: 10.2176/nmc.cr2012-0256, PMID: 24097092 PMC4533429

[23] Lewis B Kwan E Enzmann D. DSA evaluation of the STA-MCA bypass. Neuroradiology. (1984) 26:209–12. doi: 10.1007/BF00342415 6377117

[24] Awano T Sakatani K Yokose N Kondo Y Igarashi T Hoshino T. Intraoperative EC-IC bypass blood flow assessment with indocyanine green angiography in moyamoya and non-moyamoya ischemic stroke. World Neurosurg. (2010) 73:668–74. doi: 10.1016/j.wneu.2010.03.027, PMID: 20934154

[25] Amin-Hanjani S Du X Mlinarevich N Meglio G Zhao M Charbel FT. The cut flow index: an intraoperative predictor of the success of extracranial-intracranial bypass for occlusive cerebrovascular disease. Neurosurgery. (2005) 56:75–85. doi: 10.1227/01.NEU.0000143032.35416.41, PMID: 15799795

[26] Cui H Wang Y Yin Y Wan J Fei Z Gao W. Role of intraoperative microvascular Doppler in the microsurgical management of intracranial aneurysms. J Clin Ultrasound. (2011) 39:27–31. doi: 10.1002/jcu.20751 20949570

